# Transcriptional repression of SIRT1 by protein inhibitor of activated STAT 4 (PIAS4) in hepatic stellate cells contributes to liver fibrosis

**DOI:** 10.1038/srep28432

**Published:** 2016-06-21

**Authors:** Lina Sun, Zhiwen Fan, Junliang Chen, Wenfang Tian, Min Li, Huihui Xu, Xiaoyan Wu, Jing Shao, Yaoyao Bian, Mingming Fang, Yong Xu

**Affiliations:** 1 State Key Laboratory of Reproductive Medicine, Department of Pathophysiology, Key Laboratory of Cardiovascular Disease and Molecular Intervention, Nanjing Medical University, Nanjing, China; 2 Department of Pathology and Pathophysiology, School of Biology and Basic Medical Sciences, Soochow University, Suzhou, China; 3 Department of Pathology, Nanjing Drum Tower Hospital, the Affiliated Hospital of Nanjing University Medical School, Nanjing, China; 4 Department of Pathophysiology, School of Basic Medical Sciences, Jiangnan University, Wuxi, China; 5 School of Nursing, Nanjing University of Chinese Medicine, Nanjing, China; 6 School of Basic Medical Sciences, Nanjing University of Chinese Medicine, Nanjing, China; 7 Department of Nursing, Jiangsu Jiankang Vocational University, Nanjing, China

## Abstract

Interstitial fibrosis represents a key pathological process in non-alcoholic steatohepatitis (NASH). In the liver, fibrogenesis is primarily mediated by activated hepatic stellate cells (HSCs) transitioning from a quiescent state in response to a host of stimuli. The molecular mechanism underlying HSC activation is not completely understood. Here we report that there was a simultaneous up-regulation of PIAS4 expression and down-regulation of SIRT1 expression accompanying increased hepatic fibrogenesis in an MCD-diet induced mouse model of NASH. In cultured primary mouse HSCs, stimulation with high glucose activated PIAS4 while at the same time repressed SIRT1. Over-expression of PIAS4 directly repressed SIRT1 promoter activity. In contrast, depletion of PIAS4 restored SIRT1 expression in HSCs treated with high glucose. Estrogen, a known NASH-protective hormone, antagonized HSC activation by targeting PIAS4. Lentivirus-mediated delivery of short hairpin RNA (shRNA) targeting PIAS4 in mice ameliorated MCD diet induced liver fibrosis by normalizing SIRT1 expression *in vivo*. PIAS4 promoted HSC activation in a SIRT1-dependent manner *in vitro*. Mechanistically, PIAS4 mediated SIRT1 repression led to SMAD3 hyperacetylation and enhanced SMAD3 binding to fibrogenic gene promoters. Taken together, our data suggest SIRT1 trans-repression by PIAS4 plays an important role in HSC activation and liver fibrosis.

Accompanying changes in life style and dietary choices there is a growing global pandemic of obesity and related metabolic disorders, which include non-alcoholic steatohepatitis or NASH^1^. One of the devastating consequences of NASH is extensive interstitial fibrosis in the liver^2^. Although fibrogenesis is considered a adaptive host defense response to various injurious signals, chronic and deregulated hepatic fibrosis if left attended to can transition into irreversible cirrhosis and hepatocellular carcinoma, which account for the majority of deaths in patients with liver diseases^3^,^4^. Despite of decades of vigorous clinical and basic research efforts, the molecular mechanism underlying liver fibrosis is not completely understood.

Hepatic stellate cells (HSCs) are believed to play an essential role in liver fibrosis^5^. Trans-differentiation of quiescent HSCs to an activated state represents a key process in NASH-associated fibrosis. During NASH pathogenesis, HSCs are exposed to a plethora of nutrients (e.g., high concentrations of glucose) and become activated gaining the ability to proliferate and synthesize extracellular matrix (ECM) proteins^6^. Therefore, HSC activation parallels an overhaul of its transcriptional program featuring a robust up-regulation of pro-fibrogenic ECM genes such as collagen type I (*Col1a1/Col1a2*) and alpha smooth muscle actin (*Acta2*). Several sequence-specific transcription factors contribute to the reprogramming of HSC transcriptome, among which SMAD3 is the most extensively investigated.

SMAD3 has long been documented as a mediator of the signaling pathway downstream of transforming growth factor (TGF-β), the preeminent pro-fibrogenic humoral factor in the liver^7^,^8^,^9^. SMAD3 activity can be modulated by its post-translational modifications. Serine phosphorylation, for instance, is essential for SMAD3 dimmerization and nuclear translocation^10^. Lysine acetylation of SMAD3 enhances its binding to target DNA^11^,^12^. On the other hand, SMAD3 deacetylation by SIRT1, a class III protein deacetylase, has been implicated in kidney fibrosis^13^. Previously, our laboratory has identified a pathway in which the SUMO E3 ligase PIAS4 represses SIRT1 transcription to promote cancer metastasis^14^,^15^. Here we report that PIAS4 mediates SIRT1 repression in cultured HSCs exposed to high glucose and in a mouse model of NASH-associated liver fibrosis. PIAS4 promotes HSC activation and liver fibrosis by stimulating SMAD3 acetylation and target binding in a SIRT1-dependent manner. Therefore, targeting PIAS4 might facilitate the development of novel therapeutic solutions against liver fibrosis in the context of NASH.

## Results

### PIAS4 up-regulation and SIRT1 down-regulation accompany steatosis-associated liver fibrosis in mice

**Figure 1:**
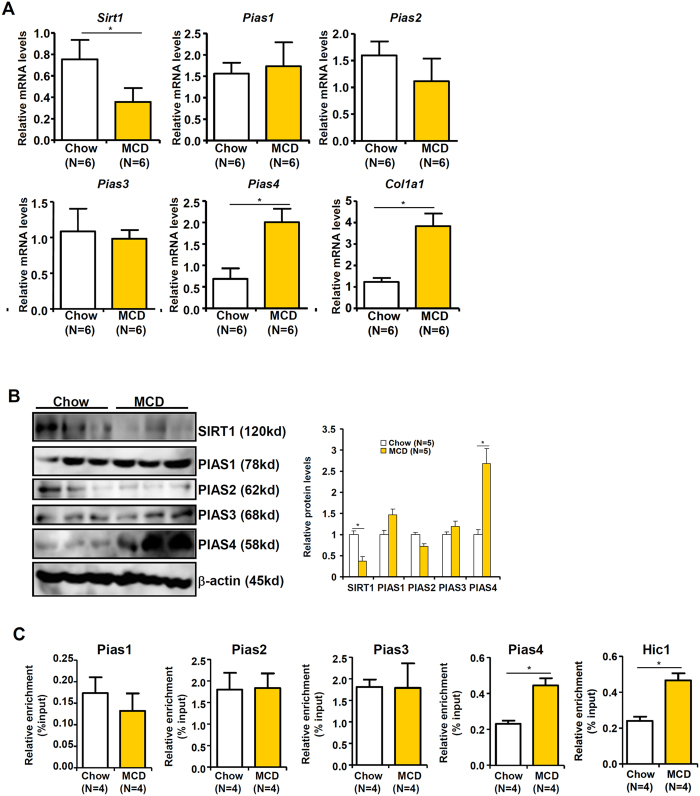
PIAS4 up-regulation and SIRT1 down-regulation accompany steatosis-associated liver fibrosis in mice. Male *db/db* mice were fed on the MCD diet or a control diet (chow) for 4 weeks. (**A**,**B**) Expression of SIRT1 and PIAS4 was examined by qPCR (**A**) and Western blotting (**B**). (**C**) Binding of PIAS proteins to the SIRT1 promoter was evaluated by ChIP.

We evaluated the involvement of PIAS4 in liver fibrosis employing a classic model of NASH wherein *db/db* mice were fed on a methionine-and-choline deficient (MCD) diet for 4 weeks^16^. Quantitative PCR (Fig. 1A) and Western blotting (Fig. 1B) analyses found that accompanying up-regulation of fibrogenic proteins such as collagen type I (*Col1a1*), there was a concomitant increase in PIAS4 expression and a decrease in SIRT1 expression in the livers of MCD-fed mice. Meanwhile, other PIAS family members including PIAS1, PIAS2, and PIAS3 did not show appreciable changes in the livers of MCD-fed mice compared to the control mice. Chromatin immunoprecipitation (ChIP) assay indicated that there was enhanced occupancy of PIAS4, but not that of PIAS1, PIAS2, or PIAS3, on the SIRT1 promoter in the livers of MCD-fed mice compared to the control mice (Fig. 1C); meanwhile, binding of the SIRT1 repressor HIC1, whose activity is regulated PIAS4-mediated SUMOylation, was up-regulated in the livers of MCD-fed mice. Collectively, these data suggest that PIAS4 might repress SIRT1 expression during liver fibrogenesis in the context of NASH pathogenesis.

### PIAS4 mediates transcriptional repression of SIRT1 by high glucose in hepatic stellate cells

**Figure 2:**
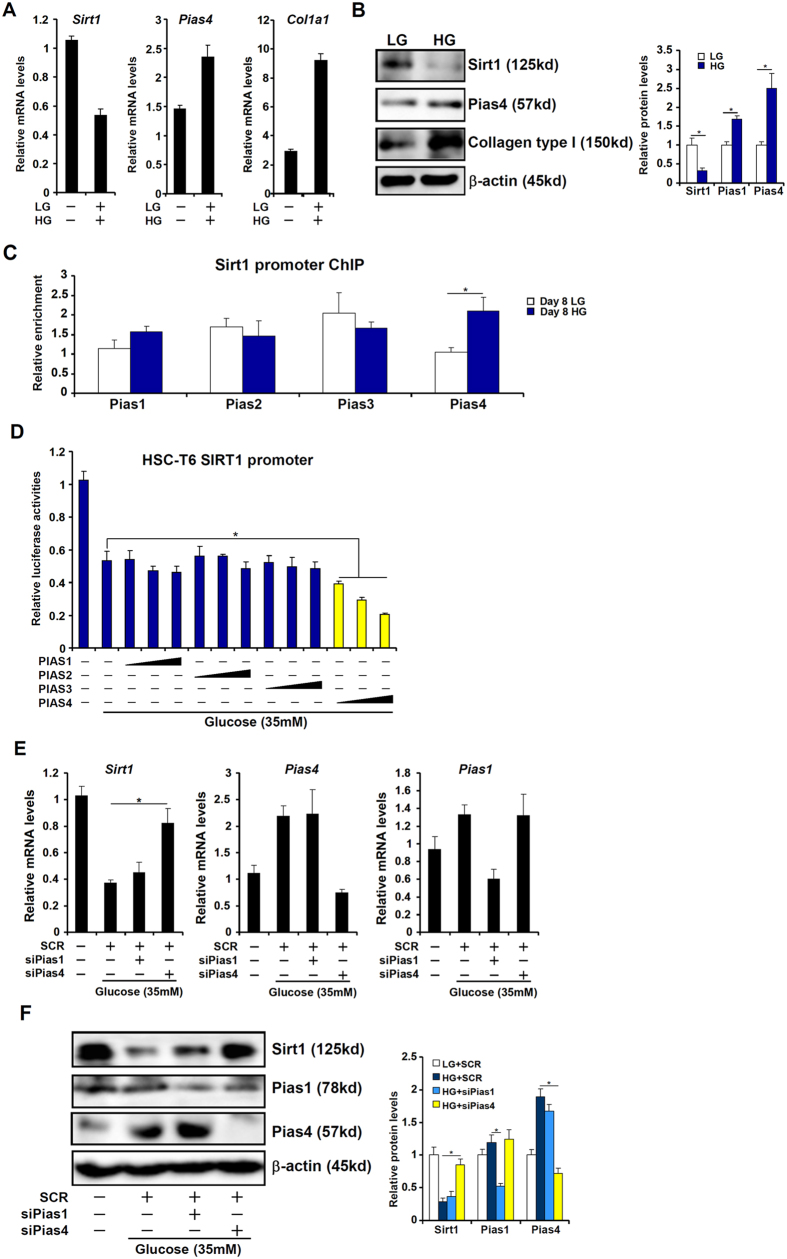
PIAS4 mediates transcriptional repression of SIRT1 by high glucose in hepatic stellate cells. (**A**–**C**) Primary mouse hepatic stellate cells were treated with glucose (35 mM) or low glucose (5.5 mM). mRNA and protein levels were measured by qPCR (**A**) and Western (**B**). (**C**) PIAS binding to the SIRT1 promoter was examined by ChIP. (**D**) A SIRT1 promoter-luciferase construct was transfected into HSC-T6 cells along with indicated PIAS expression constructs followed by treatment with high glucose for 24 hours. Luciferase activities were normalized to protein concentration and GFP fluorescence for transfection efficiency and expressed as relative luciferase activity compared to the control group. (**E**,**F**) Primary hepatic stellate cells were transfected with indicated siRNAs followed by treatment with glucose. mRNA (**E**) and protein (**F**) levels of SIRT1 were measured by qPCR and Western.

Hepatic stellate cells (HSCs) are a major source of liver fibrogenesis^5^. On the other hand, high concentrations of glucose, a risk factor for NASH pathogenesis, have been shown to promote HSC activation^17^. Therefore we hypothesized that PIAS4 might facilitate glucose-induced HSC activation by repressing SIRT1 transcription. We first titrated the response of HSCs to different concentrations of glucose starting at 5.5 mM. As shown in Fig. S1, glucose up-regulated the expression of PIAS4 while down-regulated the expression of SIRT1 in a concentration-dependent manner but peaked at 35 mM; there was no additional increase in PIAS4 expression or decrease in SIRT1 expression when glucose concentration was raised higher to 55 mM. We therefore chose 35 mM glucose for the remainder of the experiments. Treatment with high glucose (35 mM, HG) led to an up-regulation of PIAS4 and a down-regulation of SIRT1 in both primary mouse stellate cells (Fig. 2A,B) and an immortalized stellate cell line (HSC-T6, Fig. S2A,B) compared to cells cultured in low-glucose (LG) media. In addition, PIAS4 binding to the SIRT1 promoter was augmented in response to high glucose (Figs 2C and S2C). Further, we found that estradiol, a female hormone well documented to suppress HSC activation and liver fibrogenesis^18^, attenuated HG-induced augmentation of PIAS4 expression (Fig. S3A) and SIRT1 promoter binding (Fig. S3B). Next, we transfected different PIAS expression constructs along with a SIRT1 promoter construct into HSC-T6 cells and the data showed that only PIAS4 over-expression down-regulated SIRT1 promoter activity in the presence of high glucose indicating that PIAS4 may indeed suppress SIRT1 expression in HSCs at the transcriptional level (Fig. 2D). Depletion of PIAS4, but not PIAS1, with siRNA restored SIRT1 expression in primary (Fig. 2E,F) and immortalized (Figs S4A and S4B) HSCs despite the presence of high glucose. Together, these data strongly support a model in which PIAS4 mediates transcriptional repression of SIRT1 by high glucose in hepatic stellate cells.

### PIAS4 knockdown restores SIRT1 expression and alleviates liver fibrosis in mice

**Figure 3:**
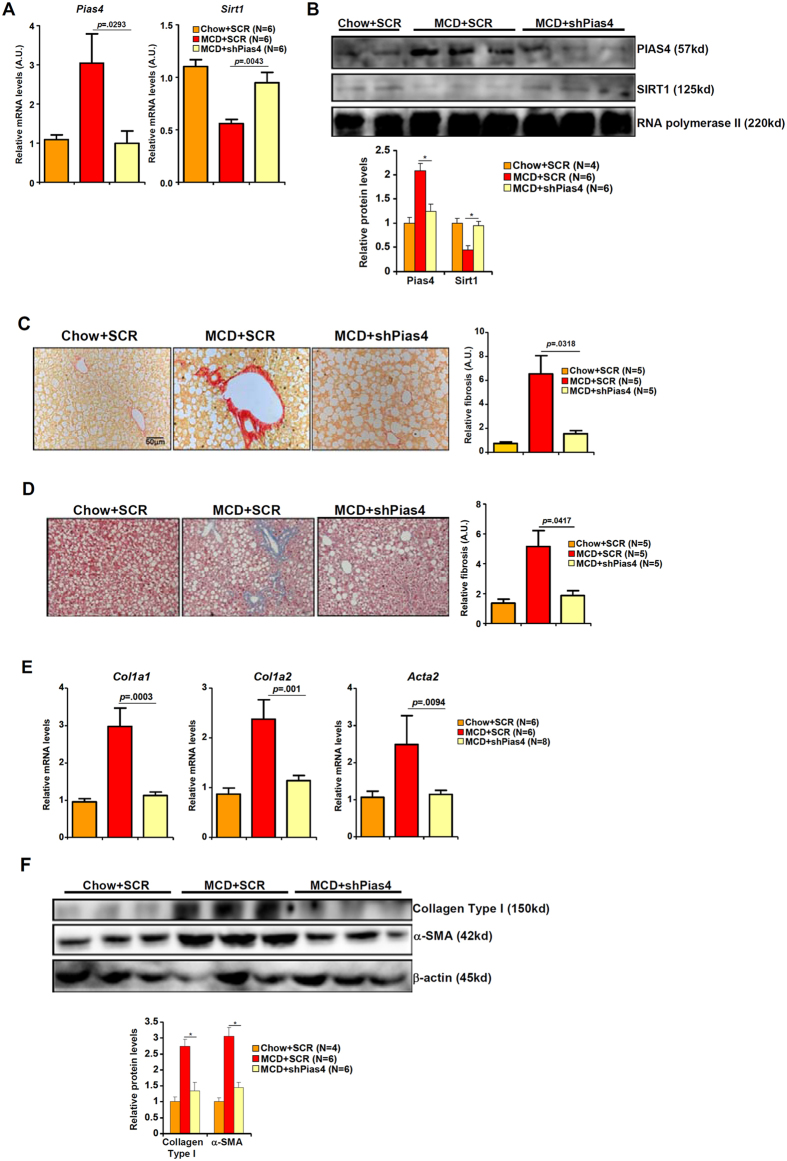
PIAS4 knockdown restores SIRT1 expression and alleviates liver fibrosis in mice. Male *db/db* mice were fed with indicated diets for 4 weeks. Silencing of PIAS4 was mediated by lentivirus as described under Methods. (**A**,**B**) Expression of PIAS4 and SIRT1 was measured by qPCR (**A**) and Western (**B**). (**C**,**D**) Paraffin embedded liver sections were stained with picrosirius red (**C**) or Masson’s trichrome (**D**). (**E**,**F**) Expression of fibrogenic proteins was measured by qPCR (**E**) and Western (**F**).

Next, we attempted to explore the possibility that PIAS4 knockdown might restore SIRT1 expression and as a result dampen liver fibrogenesis in a mouse model of NASH. Compared to MCD-fed mice receiving a control shRNA (SCR), lentivirus-mediated delivery of short hairpin RNA targeting PIAS4 (shPias4) alleviated steatotic injury as demonstrated by ALT levels (Fig. S5A) and H&E staining of inflammatory infiltrates (Fig. S5B). Consistently, PIAS4 knockdown attenuated hepatic inflammation in MCD-fed mice as evidenced by the down-regulation of several pro-inflammatory mediators (Fig. S6). Importantly, qPCR (Fig. 3A) and Western blotting (Fig. 3B) analyses showed that PIAS4 depletion normalized SIRT1 expression in the livers of MCD-fed mice. This was consistent with a decrease in the occupancy of HIC1 on the SIRT1 promoter (Fig. S5C). Picrosirius red (Fig. 3C) and Masson’s trichrome (Fig. 3D) stainings indicated that following PIAS4 knockdown there was much less intensive fibrosis in the livers of MCD-fed mice. Giving further support to the conclusion that PIAS4 depletion down-regulated liver fibrosis in mice was the observation that expression levels of several pro-fibrogenic marker genes including collagen type I (*Col1a1*, *Col1a2*) and alpha smooth muscle actin (*Acta2*) were markedly decreased in MCD-fed mice receiving shPias4 lentivirus than those receiving SCR lentivirus (Fig. 3E,F). Therefore, PIAS4 might play a critical role in promoting steatosis-associated fibrogenesis *in vivo* likely through repressing SIRT1 transcription.

### PIAS4 knockdown suppresses SMAD3 acetylation and target binding in mice

**Figure 4:**
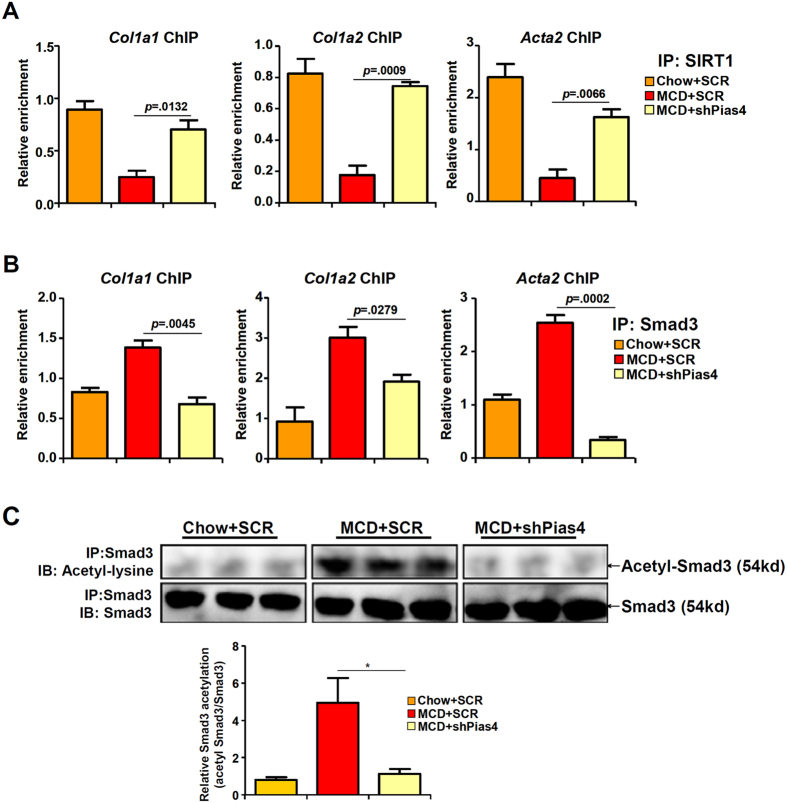
PIAS4 knockdown suppresses SMAD3 acetylation and target binding in mice. Male *db/db* mice were fed with indicated diets for 4 weeks. Silencing of PIAS4 was mediated by lentivirus as described under Methods. (**A**,**B**) Binding of SIRT1 (**A**) or SMAD3 (**B**) to fibrogenic gene promoters was examined by ChIP. (**C**) Liver homogenates were immunoprecipitated with anti-SMAD3 and the precipitated immune complex (eluate) was separated by SDS-PAGE gel electrophoresis. Western blotting was performed with indicated antibodies.

SMAD3 is a sequence-specific transcription factor essential for HSC activation and liver fibrogenesis^19^. SMAD3 can be deacetylated by SIRT1, a process contributing to the inhibition of SMAD3 activity^13^. Indeed, SIRT1 binding to SMAD3 target promoters was down-regulated in the livers of MCD mice in parallel to increased SMAD3 binding to its target genes as evidenced by ChIP assays (Fig. 4A,B). PIAS4 knockdown, however, restored SIRT1 binding to SMAD3 target promoters while simultaneously suppressed SMAD3 binding. Meanwhile, SMAD3 became hyper-acetylated in the livers of MCD-fed mice but PIAS4 knockdown rendered SMAD3 hypo-acetylated consistent with the decrease in liver fibrogenesis (Fig. 4C). Thus, we concluded that PIAS4 might contribute to liver fibrosis by modulating SIRT1-depenent SMAD3 acetylation.

### PIAS4 promotes fibrogenesis in hepatic stellate cells in a SIRT1-dependent manner

**Figure 5:**
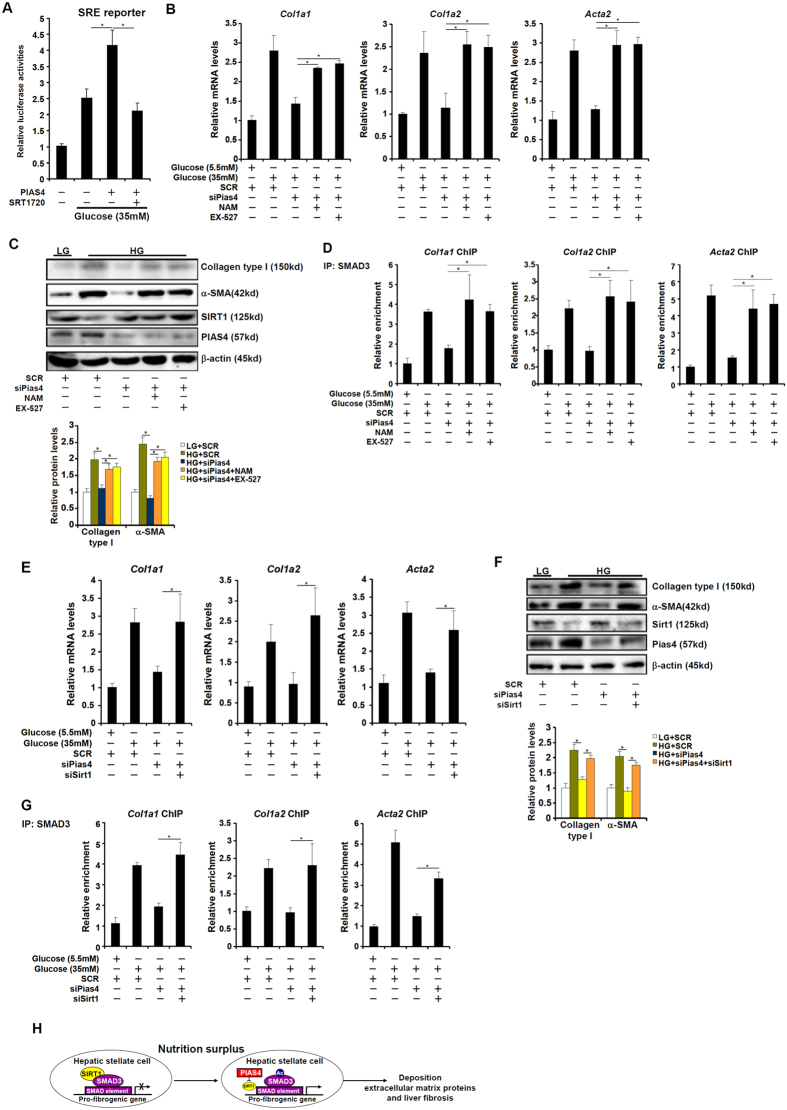
PIAS4 promotes fibrogenesis in hepatic stellate cells in a SIRT1-dependent manner. (**A**) A SMAD reporter (SRE) was transfected into HSC-T6 cells with or without PIAS4 expression construct followed by treatment with high glucose and SRT1720. Luciferase activities were normalized to protein concentration and GFP fluorescence for transfection efficiency and expressed as relative luciferase activity compared to the control group. (**B**–**D**) Primary hepatic stellate cells were transfected with indicated siRNAs followed by treatment with high glucose, NAM, and/or EX-527. Levels of fibrogenic proteins were measured by qPCR (**B**) and Western (**C**). (**D**) Binding of SMAD3 to pro-fibrogenic genes was determined by ChIP. (**E**–**G**) Primary hepatic stellate cells were transfected with indicated siRNAs followed by treatment with high glucose. Levels of fibrogenic proteins were measured by qPCR (**E**) and Western (**F**). (**G**) Binding of SMAD3 to pro-fibrogenic genes was determined by ChIP. (**H**) A schematic model depicting the regulation of HSC activation by PIAS4.

Finally, we tackled the mechanism by which PIAS4 regulates fibrogenesis in hepatic stellate cells. High glucose stimulated the activity of SMAD3 as measured by a reporter gene under the control of tandem repeats of SMAD response element (SRE) in HSC-T6 cells while over-expression of PIAS4 further enhanced the activation by glucose; pre-treatment of SRT1720, a specific SIRT1 agonist^20^, abolished the effect of PIAS4 suggesting that PIAS4 might rely on SIRT1 to regulate SMAD3 activity (Fig. 5A). Next, we used two strategies to verify whether the ability of PIAS4 to mediate glucose-induced fibrogenesis might depend on SIRT1. Pre-treatment with NAM or EX-527, two different SIRT1 antagonists, completely negated the effect of PIAS4 silencing on the production of fibrogenic genes (Figs 5B,C and S7A). On the other hand, PIAS4 knockdown repressed SMAD3 binding on the collagen type I gene promoters, which was normalized by SIRT1 inhibition (Figs 5D and S7B). Similarly, simultaneous knockdown of PIAS4 and SIRT1also restored high glucose-induced synthesis of fibrogenic genes (Figs 5E,F and S8A) and SMAD3 binding to collagen type I gene promoters (Figs 5G and S8B) even in the absence of PIAS4. These data are also consistent with the status of SMAD3 acetylation: while PIAS4 silencing dampened SMAD3 acetylation, either SIRT1 inhibition or SIRT1 knockdown rescued SMAD3 acetylation levels (Fig. S9A,B). In summary, our data suggest that PIAS4 regulates fibrogenesis in cultured hepatic stellate cells by repressing SIRT1 expression.

## Discussion

Transcriptional regulation highlights the progress of liver fibrosis during NASH pathogenesis^21^,^22^. Recent advances in deep-sequencing techniques have confirmed a relationship between genome-wide changes in liver transcriptome and disease stages in patients with chronic liver diseases^23^,^24^,^25^. Here we report that transcriptional repression of SIRT1 by PIAS4 contributes to liver fibrosis in response to excessive glucose (Fig. 5H).

We show here that in mice fed on a pro-fibrogenic MCD diet, PIAS4 directly binds to the SIRT1 promoter and represses SIRT1 transcription. Furthermore, PIAS4 also mediates the trans-repression of SIRT1 in cultured HSCs exposed to high concentrations of glucose. It is noteworthy that we have used an over-simplified cell model (glucose stimulation) to extrapolate the animal experiments. As such these data must be interpreted with extreme precaution as they only reflect a small part of a very complicated pathophysiological process. In addition to glucose, several other nutrients including free fatty acids and fructose when present in excess have been shown to promote fibrosis in the context NASH^26^,^27^. On the other hand, exposure to high glucose can induce the accumulation of reactive oxygen species (ROS) and trigger ER stress response, both of which can promote HSC activation even after glucose withdrawal^28^,^29^. It is highly likely that the combination of several different nutrients and their derivatives collectively promotes HSC activation. Whether and if so how PIAS4-mediated SIRT1 trans-repression fits into this scenario remains to be determined and should be exhaustively explored in future studies using more representative and more accurate cell models.

The current dataset suggests that in response to glucose stimulation, PIAS4-mediated SIRT1 repression leads to deacetylation of SMAD3, which in turn switches on the pro-fibrogenic transcriptional program. Several caveats exist for this proposal. First, since SIRT1 has been shown to suppress epithelial-to-mesenchymal transition (EMT), a key process contributing to fibrosis^14^,^15^,^30^, then our observation could be construed as reduced EMT process independent of SMAD3 deacetylation. A second explanation could be derived from the fact that several components of the TGF-β signaling pathway including SMAD3 are SUMOylated^31^,^32^,^33^. Therefore, PIAS4 may directly fine-tune pro-fibrogenic transcription by SUMOylating SMAD3 in parallel to its indirect effect on SMAD3 by repressing SIRT1. PIAS4-mediated SUMOlyation of SMAD3 and SIRT1-mediated deacetylation of SMAD3, however, are not mutually exclusive as it has been previously shown that PIAS4 and SIRT1 can co-modify and co-regulate the activity of transcription factor HIC1^34^. Finally, the conclusiveness of our data is further compromised by the choice of the MCD-fed *db/db* mouse model. Although fibrosis occurs relatively fast in this model (4~6 weeks as opposed to 10~12 weeks in the high fat diet model), these mice lose significant weight, which is contrary to typical human NASH pathogenesis. Therefore, similar data based on alternative (and ideally several different) NASH animal models should be sought before the relevance of the current finding to human pathology can be convincingly established.

We show here that lentivirus mediated PIAS4 knockdown in MCD-fed mice alleviates liver fibrosis, which could be explained by PIAS4 mediated SIRT1 trans-repression in HSCs. Alternatively, pro-inflammatory cells and circulating mediators are known to promote HSC activation and liver fibrosis^35^. Since there was a decrease in inflammatory infiltrates (Fig. S5B) and a parallel decrease in pro-inflammatory mediators (Fig. S6) in the liver following PIAS4 depletion, an equally plausible explanation could be that down-regulation of liver fibrosis might be secondary to inhibition of liver inflammation. Indeed, a number of recent studies using cell co-culture models have demonstrated that HSCs exposed to paracrine signals emitted from “inflamed” hepatocytes or macrophages become activated more rapidly^36^,^37^. In order to resolve this issue and assign a more precise, cell-autonomous role of PIAS4 in liver fibrosis, future study should take advantage of the Cre-Flox system to specifically delete PIAS4 in different hepatic cells.

In conclusion, we present evidence that PIAS-mediated transcriptional repression of SIRT1 contributes to HSC activation and liver fibrosis in the context of NASH pathogenesis. More investigations are warranted to further clarify the role of PIAS4 in this process before the effort of targeting PIAS4 for therapeutic purposes can be moved forward.

## Methods

### Cell culture

Immortalized rat hepatic stellate cells (HSC-T6, ATCC) were maintained in DMEM supplemented with 10% fetal bovine serum (FBS). Primary murine hepatic stellate cells were isolated as described previously^38^.

### Plasmids, transfection, and reporter assay

PIAS expression constructs^14^, Ubc9 expression construct^15^, SIRT1 promoter construct^39^, collagen type I gene promoter constructs^40^, and α-SMA gene promoter construct^8^ have been previously described. Transient transfections were performed with Lipofectamine 2000 (Invitrogen). Luciferase activities were assayed using a luciferase reporter assay system (Promega). All experiments were repeated at least three times.

### Animals

All animal protocols were approved by the NJMU Intramural Ethics Committee on Animal Studies and adhere to the criteria outlined in the “Guide for the Care and Use of Laboratory Animals”. To induce steatosis, 6~8 week-old male *db/db* mice were fed on an MCD diet (A02082002B, Research Diets) for 4 consecutive weeks. In certain experiments, these mice were injected via tail vein purified lentiviral particles (1 × 10^9^ MOI) that carry short hairpin RNA (shRNA) targeting PIAS4 (GTGCTGTACGGGAAGTACTT) or scrambled shRNA (SCR) every 10 days for the duration of the experiments.

### Protein Extraction, and Western Blot

Whole cell lysates were obtained as previously described^41^. Western blot analyses were performed with anti-collagene type I (Rockland), anti-α-SMA, anti-β-actin, anti-PIAS4 (Sigma), anti-PIAS1, anti-PIAS2, anti-PIAS3, anti-RNA polymerase II (Santa Cruz), anti-acetyl lysine (Cell Signaling Tech), anti-SMAD3, and anti-SIRT1 (Abcam) antibodies.

### RNA Isolation and Real-time PCR

RNA was extracted with the RNeasy RNA isolation kit (Qiagen). Reverse transcriptase reactions were performed as previously described using a SuperScript First-strand Synthesis System (Invitrogen)^9^. Primers and Taqman probes used for real-time reactions were purchased from Applied Biosystems.

### Chromatin Immunoprecipitation (ChIP)

ChIP assays were performed essentially as described before^42^ with anti-PIAS1, anti-PIAS2, anti-PIAS3 (Santa Cruz), anti-PIAS4 (Sigma), anti-SMAD3 (Cell Signaling Tech) or anti-SIRT1 (Abcam). Precipitated genomic DNA was amplified by real-time PCR with primers as previously described^43^,^44^.

### Histology

Histological analyses were performed essentially as described before^14^,^45^. Briefly, paraffin sections were stained with picrosirius red (Sigma) or Masson’s trichrome (Sigma) according to standard procedures. Pictures were taken using an Olympus IX-70 microscope. Quantification was performed using Image Pro by two investigators independently. For each slide, three different fields were assessed for positive staining. For each mouse, at least five slides were assessed. The measurements were then added and averaged and presented as relative staining compared to the control group.

### Enzyme-linked immune absorbance assay (ELISA)

Supernatants containing pro-inflammatory mediators were collected from cultured hepatocyte or liver homogenates and ELISA was performed to measure IL-1, IL-6, and MCP-1 using commercially available kits (R&D).

### Statistical analysis

One-way ANOVA with post-hoc Scheffe analyses were performed using an SPSS package. Unless otherwise specified, *p* values smaller than 0.05 were considered statistically significant (*).

## Additional Information

**How to cite this article**: Sun, L. *et al.* Transcriptional repression of SIRT1 by protein inhibitor of activated STAT 4 (PIAS4) in hepatic stellate cells contributes to liver fibrosis. *Sci. Rep.* **6**, 28432; doi: 10.1038/srep28432 (2016).

## Supplementary Material

Supplementary Information
