# Corrigendum: Transcriptional repression of SIRT1 by protein inhibitor of activated STAT 4 (PIAS4) in hepatic stellate cells contributes to liver fibrosis

**DOI:** 10.1038/srep30513

**Published:** 2016-07-27

**Authors:** Lina Sun, Zhiwen Fan, Junliang Chen, Wenfang Tian, Min Li, Huihui Xu, Xiaoyan Wu, Jing Shao, Yaoyao Bian, Mingming Fang, Yong Xu

Scientific Reports 6: Article number: 28432; 10.1038/srep28432 published online: 06 21 2016; updated: 07 27 2016.

In the original version of this Article, Jing Shao and Yaoyao Bian were incorrectly affiliated with the ‘School of Nursing, Nanjing University of Chinese Medicine, Nanjing, China’ and ‘School of Basic Medical Sciences, Nanjing University of Chinese Medicine, Nanjing, China’ respectively. The correct affiliations are listed below.

Jing Shao

School of Basic Medical Sciences, Nanjing University of Chinese Medicine, Nanjing, China

Yaoyao Bian

School of Nursing, Nanjing University of Chinese Medicine, Nanjing, China

These errors have now been corrected in the PDF and HTML versions of the Article.

