# Supplementary material for: Transcriptional repression of SIRT1 by protein inhibitor of activated STAT 4 (PIAS4) in hepatic stellate cells contributes to liver fibrosis
Source: Sci Rep. 2016 Jun 21;6:28432. doi: 10.1038/srep28432 (PMC4914937; doi:10.1038/srep28432)
Supplement: Supplementary Information [file srep28432-s1.pdf]

Lina Sun, Zhiwen Fan, Junliang Chen, Wenfang Tian, Min Li, Huihui Xu, Xiaoyan Wu, Jing Shao, Yaoyao Bian, Mingming Fang, and Yong Xu: ***Transcriptional repression of SIRT1 by protein inhibitor of activated STAT 4 (PIAS4) in hepatic stellate cells contributes to liver fibrosis***

Online supplementary material

Figures: 9

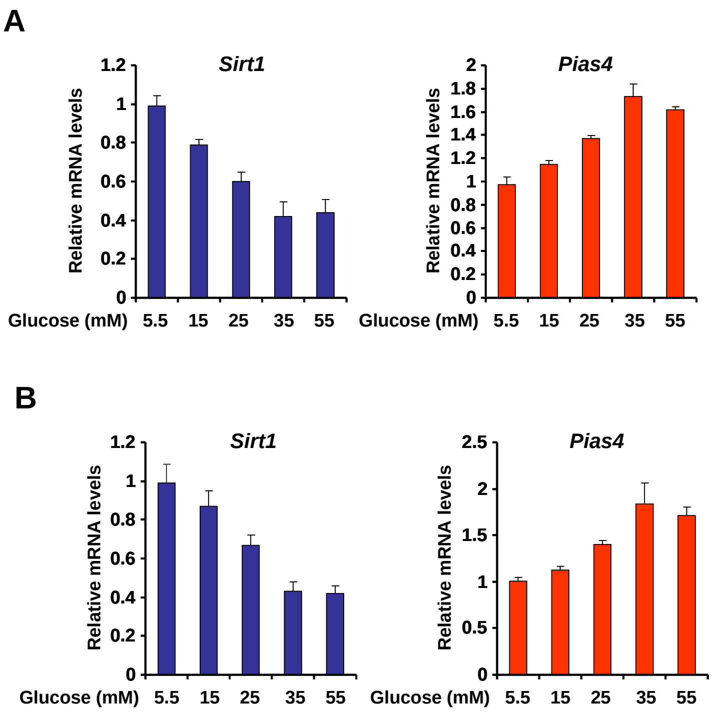

Fig.S1: (**A, B**) HSC-T6 cells (A) or primary mouse HSCs (B) were cultured in media containing different concentrations of glucose for 8 days. Expression levels of SIRT1 and PIAS4 were measured by qPCR.

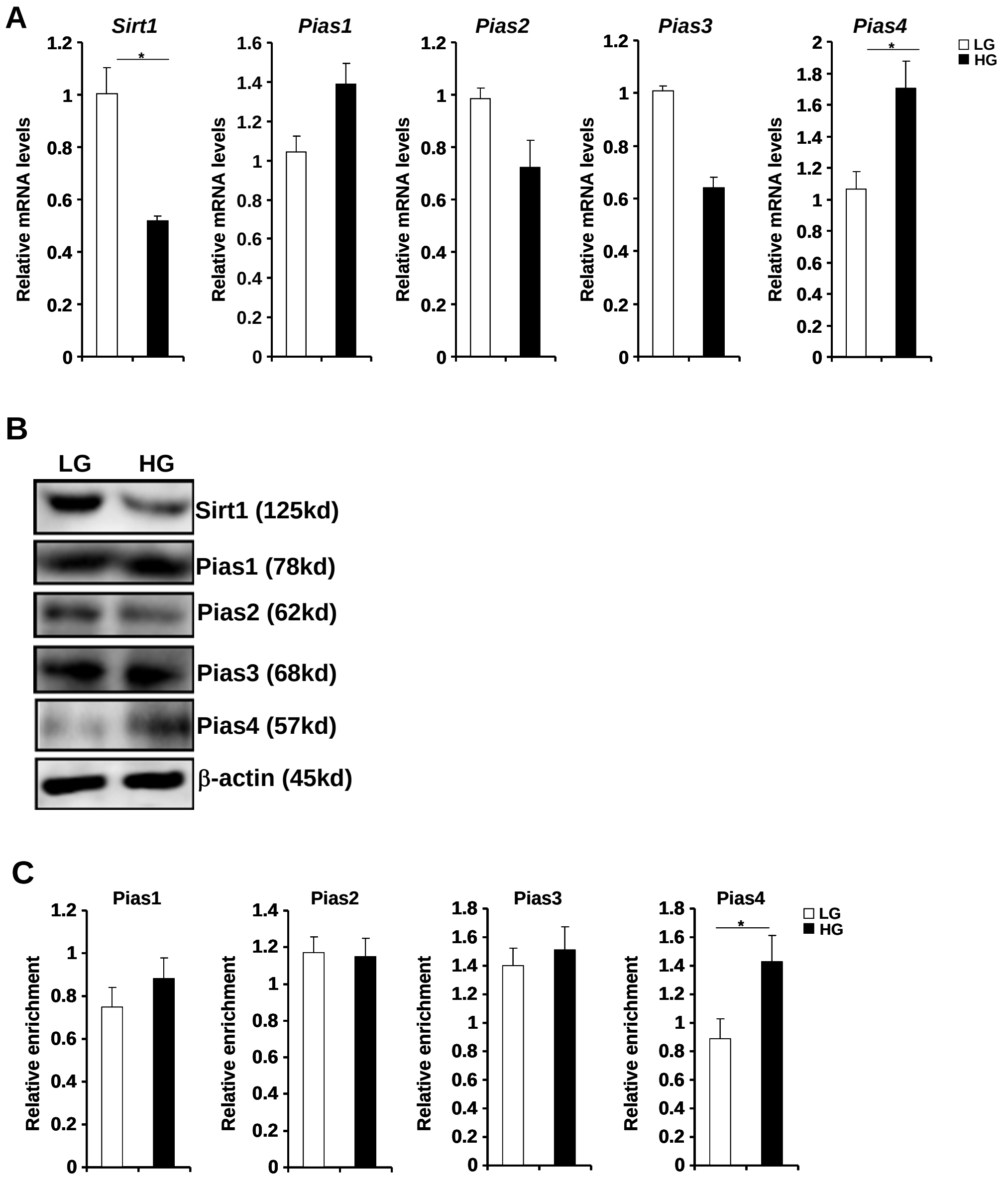

Fig.S2: (**A-C**) HSC-T6 cells were cultured in either low glucose or high glucose media for 8 days. Expression levels of SIRT1 and PIAS4 were measured by qPCR (A) and Western (B). PIAS binding to the SIRT1 promoter was examined by ChIP (C).

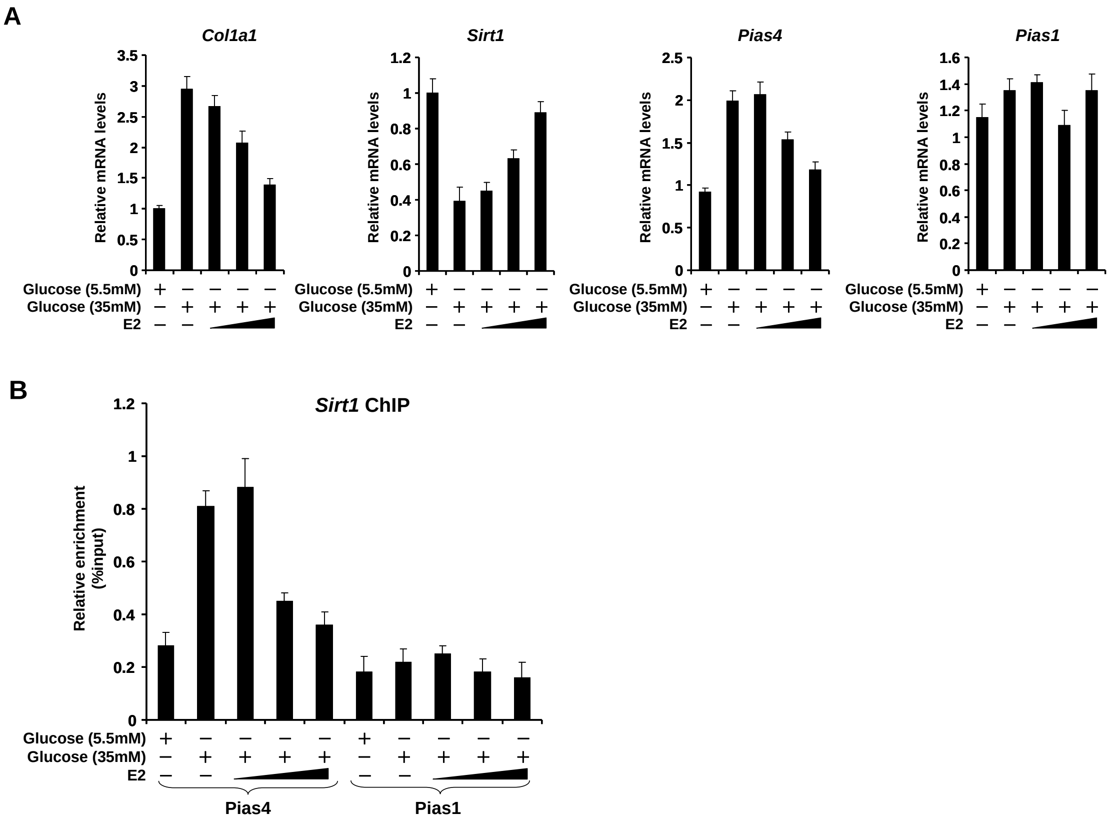

Fig.S3: (**A, B**) HSC-T6 cells were treated with glucose and/or estradiol for 24 hours. Expression of SIRT1 and PIAS proteins was measured by qPCR (A). Binding of PIAS proteins to the SIRT1 promoter was measured by ChIP (B).

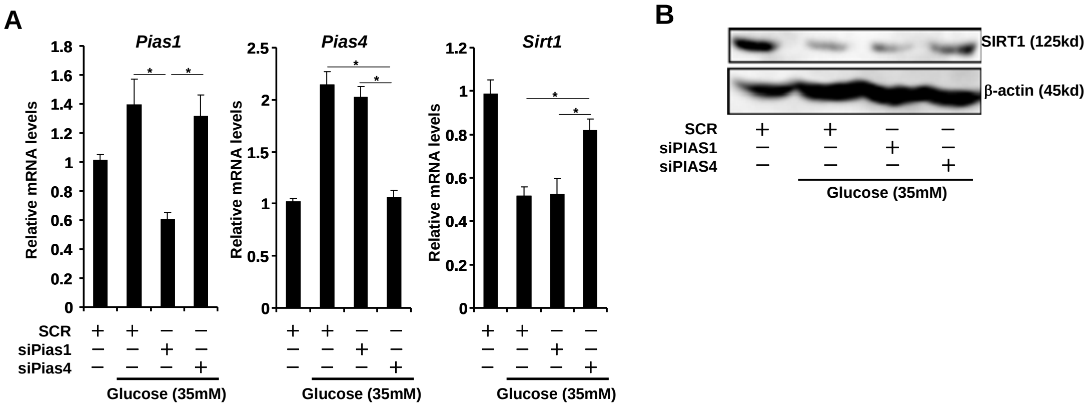

Fig.S4: (**A, B**) HSC-T6 cells were transfected with indicated siRNAs followed by treatment with high glucose. Expression of SIRT1 was measured by qPCR and Western.

A

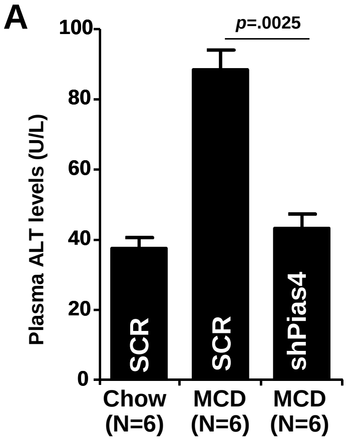

B

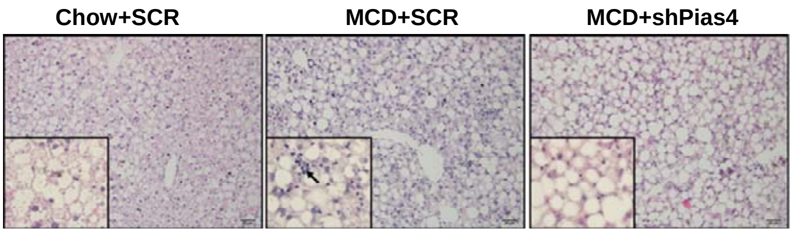

Score\Group	Chow+SCR	MCD+SCR	MCD+shPias4
Necroinflammation	1.00±0.22	4.09±1.17	1.59±0.35*

C

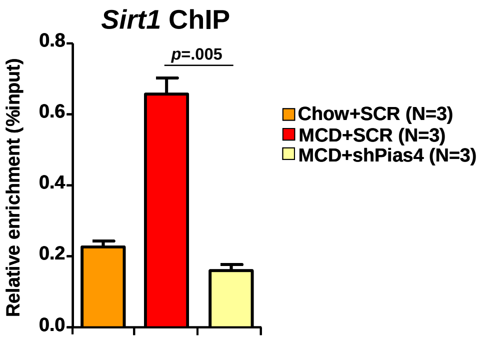

Fig.S5: Male *db/db* mice were fed with the MCD diet or control (AL) diet for 4 weeks. Silencing of PIAS4 was mediated by lentivirus as described under Methods. (**A**) Levels of ALT were measured by ELSA. (**B**) H&E staining was performed as described under Methods. *, $p < .05$ (**C**) HIC1 binding to the SIRT1 promoter was evaluated by ChIP using liver homogenates.

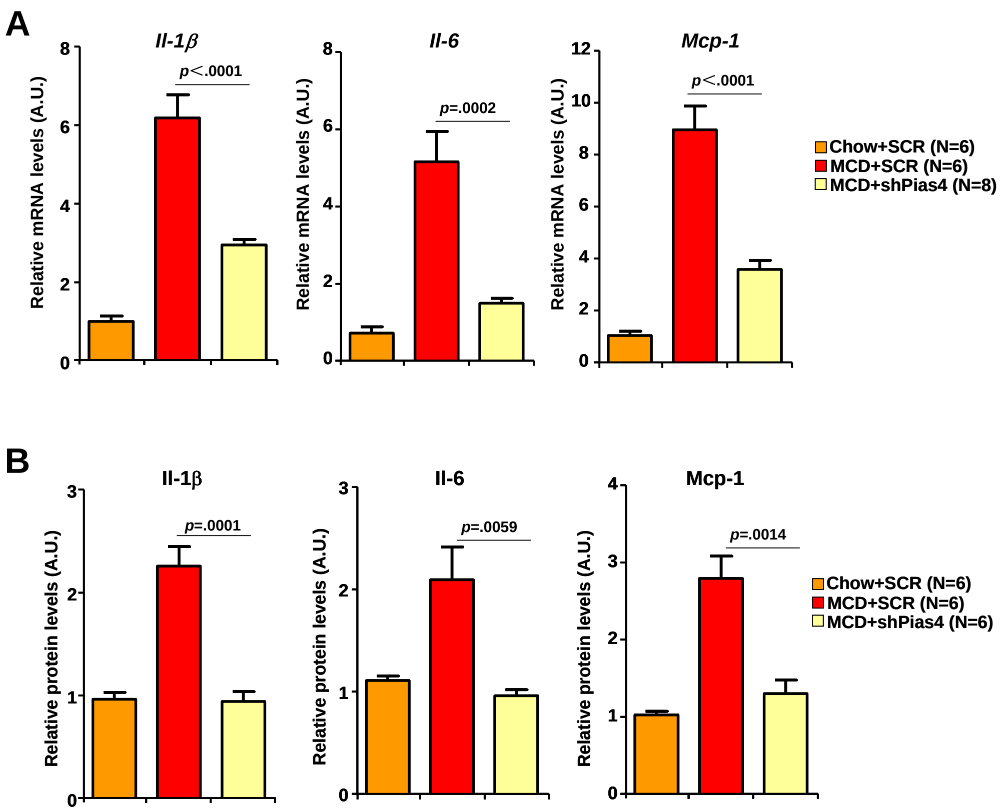

Fig.S6: Male *db/db* mice were fed with the MCD diet or control (AL) diet for 4 weeks. Silencing of PIAS4 was mediated by lentivirus as described under Methods. (**A, B**) Levels of pro-inflammatory mediators in the liver were examined by qPCR and ELISA.

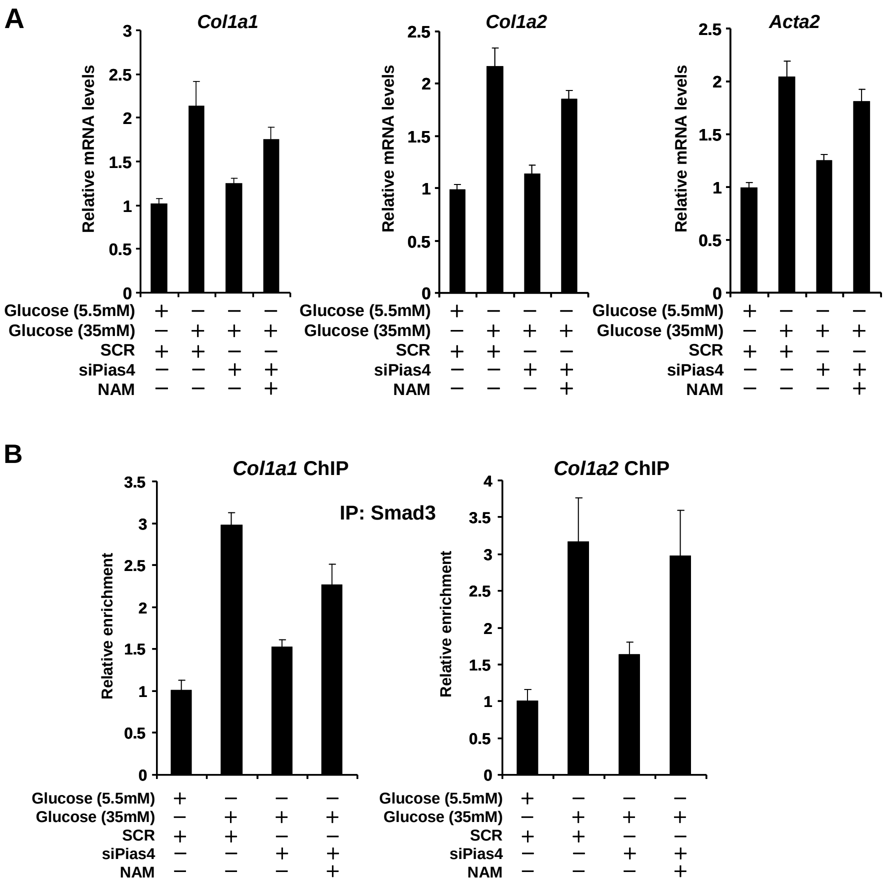

Fig.S7: (**A, B**) HSC-T6 cells were transfected with indicated siRNAs followed by treatment with high glucose and/or NAM. Expression of pro-fibrogenic genes was measured by qPCR (A). Binding of Smad3 to pro-fibrogenic gene promoters was determined by ChIP (B).

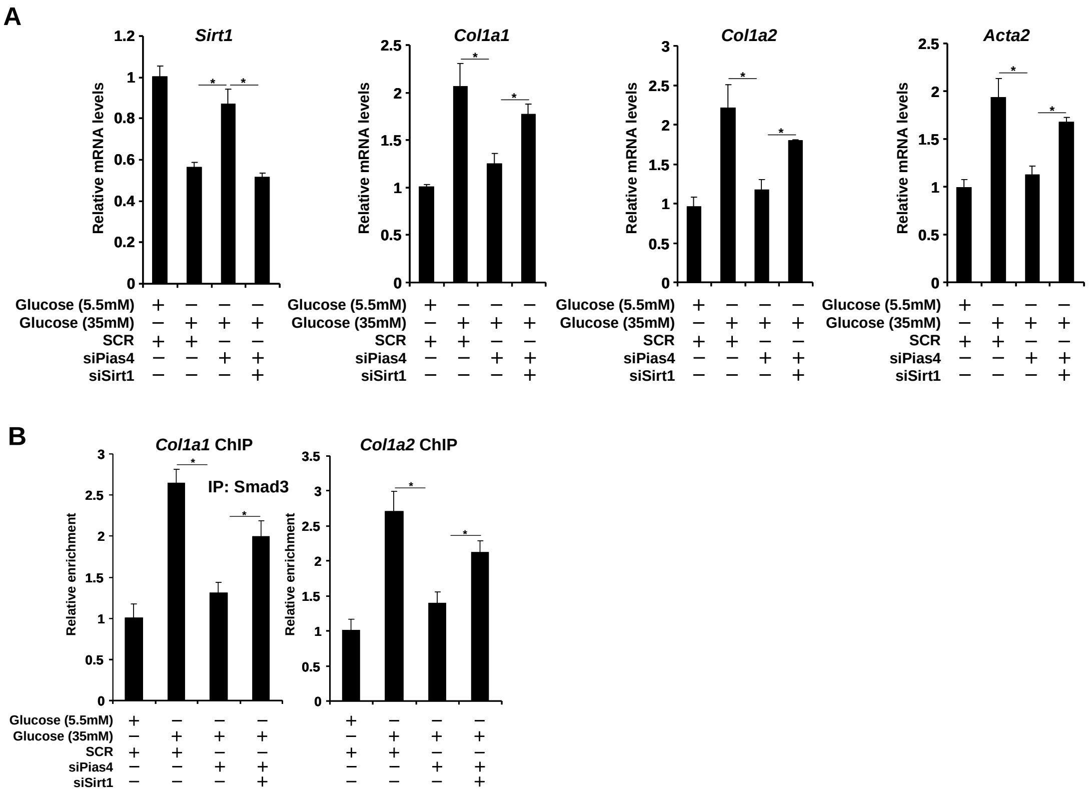

Fig.S8: (**A, B**) HSC-T6 cells were transfected with indicated siRNAs followed by treatment with high glucose. Expression of pro-fibrogenic genes was measured by qPCR (A). Binding of Smad3 to pro-fibrogenic gene promoters was determined by ChIP (B).

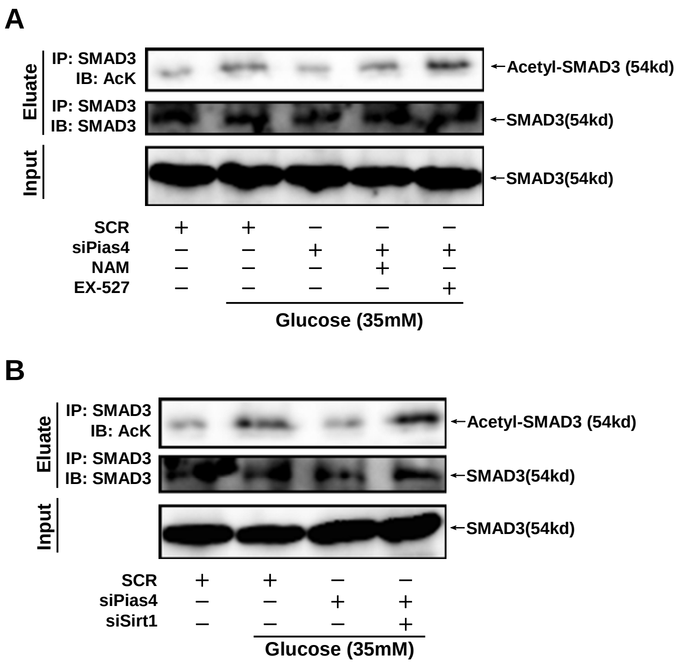

Fig.S9: (**A**) Primary hepatic stellate cells were transfected with indicated siRNAs followed by treatment with glucose, NAM, and/or EX-527. Whole cell lysates were immunoprecipitated with anti-SMAD3 and the precipitated immune complex (eluate) was separated by SDS-PAGE gel electrophoresis. Western blotting was performed with indicated antibodies. 10% of the starting material was included as input. (**B**) Primary hepatic stellate cells were transfected with indicated siRNAs followed by treatment with glucose. Whole cell lysates were immunoprecipitated with anti-SMAD3 and the precipitated immune complex (eluate) was separated by SDS-PAGE gel electrophoresis. Western blotting was performed with indicated antibodies. 10% of the starting material was included as input.
